# Dietary Factors as Triggers of Low-Grade Chronic Intestinal Inflammation in Poultry

**DOI:** 10.3390/microorganisms8010139

**Published:** 2020-01-19

**Authors:** Gabriela Cardoso Dal Pont, Morgan Farnell, Yuhua Farnell, Michael H. Kogut

**Affiliations:** 1 Department of Poultry Science, Texas A&M AgriLife Research, College Station, TX 77845, USA; mfarnell@tamu.edu (M.F.); yfarnell@tamu.edu (Y.F.); 2 Southern Plains Agricultural Research Center, USDA-ARS, College Station, TX 77845, USA; mike.kogut@usda.gov

**Keywords:** gut health, inflammatory response, intestine, chicken, broiler, immunity, immune system

## Abstract

Inflammation is the reaction of the immune system to an injury; it is aimed at the recovery and repair of damaged tissue. The inflammatory response can be beneficial to the animal since it will reestablish tissue homeostasis if well regulated. However, if it is not controlled, inflammation might lead to a chronic response with a subsequent loss of tissue function. The intestine is constantly exposed to a number of environmental triggers that stimulate inflammation and lead to a reduction in performance. The diet and dietary components constitute consistent inflammatory triggers in poultry. Dietary components, such as anti-nutritional compounds, oxidized lipids, mycotoxins, and excess of soluble fiber or protein, are all capable of inducing a low-grade inflammatory response in the intestine of broilers throughout a 5-week grow-out period. We hypothesized that dietary factor-induced chronic intestinal inflammation is a key driver of the lower performance and higher incidence of intestinal problems observed in poultry production. Therefore, this review was aimed at exploring feed-induced chronic inflammation in poultry, the constituents of the diet that might act as inflammatory triggers and the possible effects of chronic intestinal inflammation on the poultry industry.

## 1. Introduction

The poultry industry is aware that intestinal disorders reduce flock performance, increase morbidity, and increase the bacterial contamination of the meat [1]. However, less attention has been paid to the subclinical changes in the gut which might affect the systemic physiological homeostasis. We hypothesized that subclinical gut disorders lead to a chronic low-level inflammatory response in the gut, resulting in the disruption of digestive function, a constant state of oxidative stress, and poor immune competence. 

Multiple environmental factors of commercial production can trigger gut inflammation, including animal density, reused litter, intestinal pathogens such *Eimeria* sp., poor quality feed ingredients, high energy diets, and changes in feed formulation [2,3,4]. For years, the effects of these challenges to the animal gastrointestinal tract (GIT) have been controlled by antibiotics added in the feed as growth promoters (AGP) which, in addition to their antimicrobial effects, also reduce low-level inflammation [5]. However, non-AGP poultry production, demanded by the consumer pressure [6,7], has been a challenge to the industry in controlling health and maintaining performance standards [8,9,10]. Thus, it is believed that the decreased performance in AGP-free production is due to increased challenges that the GIT experiences, resulting the induction of chronic low-level gut inflammation. Therefore, sustainable antibiotic-free poultry production will be dependent upon a better understanding of gut health and its application in production systems [11]. Consequently, gut health has been a primary focus in the non-AGP era and has become one of the most used phrases in the scientific lexicon of animal production and research [12,13].

Hence, poor gut health and chronic low-level gut inflammation are important topics for optimal antibiotic-free poultry production. This review was aimed at exploring chronic gut inflammation, its causes and possible consequences. Furthermore, we summarize the dietary factors that can impair gut health and induce gut inflammation. Few studies have explored the role of feed components and their unfavorable effects on a chronic gut inflammation. Therefore, this review connects what is known in poultry to the knowledge from other species.

## 2. Intestinal Inflammation in Poultry: An Introduction

Inflammation is the primary effector mechanism of the innate immune system (IIS), whose primary function is the recovery and repair of infected and/or damaged tissue [14]. However, inflammation does cause tissue damage with the loss of functionality if poorly regulated [15]. In the intestine, this immune regulation is crucial to maintain homeostasis since the organ is continuously exposed to non-self-derived triggers, such as pathogenic microbes, food antigens, and toxins that could generate inflammation [16]. As the organ with the largest number of resident immune cells [17], homeostasis of the intestinal physiology is dependent of the complex communication and tight regulation between immune cells, cytokines, the microbiota, microbiome-derived metabolites and the host [2,18,19]. If an imbalance in this homeostasis occurs from an environmental insult (either infectious or non-infectious), a highly regulated cascade of physiological and immunological events will be activated, resulting in an inflammatory response [20].

The function and mechanisms of an inflammatory response in poultry have recently been described in detail by us in a series of reviews [2,14,21,22]. Briefly, innate immune cells (macrophages, granulocytes, dendritic cells, intestinal epithelial cells) express pattern recognition receptors (PRRs) that recognize and respond to infectious microbial constituents, as microbe-associated molecular patterns (MAMPs) [23,24,25] and to endogenous host molecules released during cell death or stress, called damage associated molecular patterns (DAMPs) [14,21,22,23,24,25]. During homeostasis, the intestinal immune tissue remains tolerant to microbiota and dietary antigens. However, during toxic insult or infection or dysbiosis, the PRRs can activate and initiate a cascade of events that induce an inflammatory response [2,14,21,22].

Recently, inflammatory phenotypes in poultry have been described as physiological, pathological, sterile and metabolic [2,14]. The term “physiological inflammation” defines the controlled inflammatory response of gut that regulates gut immunity homeostasis, preventing intestinal tissue damage [2,26]. Metabolic inflammation results from the continuous stimulation of PRRs by excess levels of dietary nutrients and/or metabolites [27,28], such as free fatty acids, carbohydrates and lipids [29]. Sterile inflammation is characterized by the absence of infection but with a low-grade response to DAMPS induced by chemical (oxidative stress), physical (microbiota components), and/or metabolic stimuli (dietary components) resulting in cell death [30]. Metabolic and sterile inflammation are typical examples of chronic low-grade inflammatory states that are likely to occur as features of modern animal production due to the high feed intake, nutrient excess, the ingredients used in the diet and overall environment that the birds are exposed to [2].

## 3. Intestinal Inflammation on Poultry Production

Gut inflammation promotes drastic alterations on intestinal architecture, resulting in leaky gut [31,32,33] and losses in production from decreasing digestibility, fluid loss and diarrhea and increased the moisture in the litter [32,33,34]. Furthermore, leaky gut leads to translocation of gut bacteria, microbial compounds, and/or antigens generating a systemic immune response [31,32,33,34]. Systemic inflammation requires energy and results in the loss of bird performance. In an experiment studying acute inflammation by lipopolysaccharide (LPS) (*Escherichia coli* 055:B5) injection, Jiang and collaborators [35] observed that the challenge decreased body weight gain (BWG) by 22%, but, just 59% of this reduction was caused by the decrease of consumption. Therefore, 41% of BWG depression was attributed to other factors, such as the immune response. 

Numerous factors influence gut immune health, including the microbiota, pathogens, host genetics, host age, hygiene, medications, and management practices as described previously [2,14,21,22]. However, one overlooked factor in the overall health of the intestinal immune system is the feed and feed ingredients [2,14]. Although the feed provided to broilers, breeders and layers is aimed for the birds to achieve as close as to 100% of their genetic potential, the feed can contain several components that challenge gut immune homeostasis [14,36]. Examples of such feed components are mycotoxins, non-digestible feed ingredients, products of lipid oxidation, and a wide range of anti-nutritional factors such as enzyme inhibitors and phytate. Thus, animals are constantly exposed to feed components that could trigger a low-grade inflammatory response. Over time, this response will lead to a chronic inflammation in the intestine that results in the reduction of intestinal function at the expense of energy in immune response and reduced performance [2,14].

The remainder of this review will concentrate on the dietary factors that are inflammatory and/or have inflammatory components. 

## 4. Feed Components and Their Impact on Intestinal Health in Poultry

A variety of feed components can have a negative impact on intestine homeostasis. In this section, we focus on the effects of some feed components on gut health.

### 4.1. Non-Starch Polysaccharides

The carbohydrates present in plants are divided into starch and non-starch polysaccharides (NSP), also called structural carbohydrates or fiber. Fibers and their components sometimes have confusing classification that depends on their chemical composition, extraction or dietary effect. Non-starch polysaccharides (NSP) were classified in three chemical groups by Bailey [37]: cellulose, non-cellulosic polysaccharides and pectin polymers. Moreover, the NPS can be classified according to their physical properties as soluble and insoluble in water [38,39]. The soluble group consists of arabinoxylans, glucans, fructans, pectins, and hemicelluloses [40] and show different roles in the diet and digesta than the insoluble. The NSP are not digestible by monogastric animals; therefore, part of them is metabolized by the microbiota [41]. However, the feed remains in the crop for a shorter time in poultry species which is not sufficient for NSP digestion [40] by commensal Lactobacilli or Streptococci as compared to wild birds [42]. Furthermore, the chicken intestinal microbiota is not as efficient as other non-ruminants in fiber fermentation [43]. Therefore, when these soluble NSP are in contact with mucus and secretion of intestinal tract, they form a jelly that covers the other ingredients called hydrocolloids. This coverage reduces feed contact with digestive secretions, enzymes and with the enterocytes and thus, decreases the availability of molecules for digestion and absorption [40]. Therefore, the increase of digesta viscosity by the hydrocolloids in broilers decreases body weight gain (BWG) and the feed conversion ratio (FCR) [44]. The increase in digesta viscosity caused by NSP escalates gastric transit, which decreases feed intake [45] and overall intestine transit time. The increase of digesta transit induces higher bacteria proliferation, especially undesirable microbes such as *Escherichia coli* and *Clostridium perfringens* [46,47]. Moreover, diets with high NSP promote higher bacterial translocation from the gut to the blood system, due to the leaky gut [48,49] that can produce systemic infection and inflammation. 

The majority of NSP present in cereals are arabinoxylans, cellulose and β-glucans [50]. Different cereals have different ratios of these NSP. Barley, wheat, rye, triticale and oats are classified as viscous cereals due to their quantity of soluble non-starch polysaccharides. Therefore, if these ingredients are used in poultry feed, especially for broilers, the effects of soluble fiber should be considered as a trigger of inflammation. Although some practices can be used to avoid the negative effects of a diet with a higher NSP content, as feed probiotics that produce phytase, lipase, xylanase and cellulases [51] add insoluble fiber to the feed and supplementation of exogenous enzymes capable of breaking NSP, such as xylanase and β-glucanase [40].

### 4.2. Oxidized Oil

Lipids are used in poultry diets mainly to increase the energy content in broilers feed, since these birds require a high metabolizable energy to express their genetic potential for rapid growth. However, the lipid profile and oil or fat quality can have an impact on the overall health of the birds while also influencing the intestine. For example, fish oil, which contains a good quantity of polyunsaturated fatty acids, has been shown to have benefits on growth performance and gut health [52]. However, fish oils are easily oxidized since oxygen affects the double bond in the fatty acids [53,54] and if oxidized, fish oil can then become hazardous to the bird. The peroxidation of lipids forms hydroxiperoxyde, an intermediate compound, and aldehydes, ketones, dicarbonyls, furans and hydrocarbons, as secondary products [55]. Toxic effects of the hydroperoxyde and the secondary products have already been known for a long time in the literature [56,57,58,59]. 

Some studies have shown that moderately oxidized oils did not result in the loss of digestibility of crude protein or ether extract [60]. However, as the intestinal mucosa is the first contact with the peroxide compounds present in the diet, it is susceptible to injuries if oil, fat, animal byproducts, rice bran, or other high fat ingredients of poor quality are used in the feed. Dibner and collaborators [56] observed that the inclusion of oxidized poultry fat in broiler feed results in the reduction of body weight, hematocrit, and the enterocyte life span, as well as increases hepatocytes proliferation and reduced the effectiveness of secretory IgA in the intestine. The increased peroxide value (POV) in the diet produced deleterious effects not just on performance but also affected gut associated lymphoid tissue (GALT) [57,58]. Furthermore, Liang and colleagues observed that the jejunum suffered oxidative stress by the oxidized oil present in the diet, which affected cytokine expression and immune cells in the tissue. Birds fed with oxidized oil showed intestinal mucosa peroxidation, and a decline in the antioxidative capacity and inadequate removal of reactive oxygen species (ROS) in the jejunum and plasma. In addition, an increase in POV in the feed reduced CD4 and CD8 molecules on the jejunum and increased the expression pro-inflammatory molecules as nuclear factor kappa B (NFκB) P50, NF-κB P65, and tumor necrosis factor-α (TNF-α). Diets with moderately oxidized fish oil, compared with fresh fish oil, showed an increase in the serum corticosterone levels, peroxidation on the liver and changes on antioxidant enzymes expression on jejunum [59]. Furthermore, oxidized fish oil reduced the expression of tight junction proteins, claudin-1 and occludin, and increased the levels of IL-22 mRNA. The decreased expression of the tight junction proteins and the increase of IL-22, a pro-inflammatory cytokine, showed a reduction in intestinal epithelial barrier integrity and an inflammatory response. 

To date, studies that evaluated the effects of oxidized oil on poultry intestine have found a profuse oxidative stress of the mucosa, a reduction of enterocytes’ half-life, impaired immune response, inflammation and the loss of the epithelial barrier. Moreover, oxidized oil had a deleterious impact on the performance of young birds, intestinal immunity and oxidative stress, even with a low oxidation level as 3.14 meqO2/kg dietary POV [58]. Therefore, to expect a bird’s high performance and healthy gut, attention should be paid to the quality of the lipid sources and ingredients high in lipid content. In addition, the method used to evaluate the oil or fat quality is important. Measurement of just the hydroxiperoxyde compounds in the ingredients could produce false interpretation, since they are intermediate compounds that will be converted to the secondary products later in the reaction [55]. Therefore, highly oxidized ingredients can be low in hydroxiperoxyde but high in the final compounds.

### 4.3. Protein

A correlation between high protein consumption and inflammatory intestinal disease in humans, such as ulcerative colitis and Crohn’s disease, has been observed [61]. Since protein is the major substrate of nitrogen to colonic microorganisms, it increases their growth and the production of short chain fatty acids (SCFA) [62]. However, protein can enhance putrefactive fermentation products [63], such as ammonia, hydrogen sulphide, amines, phenols, thiols, and indoles, which have cytotoxic, genotoxic and carcinogenic effects [64,65].

Studies with broilers have shown the influence of protein concentration and source used in the diet on gut microbiota and gut morphology. The increase of dietary indigestible protein, by rapeseed meal inclusion in the feed, for example, reduced volatile fatty acid concentration in the ceca, increased protein fermentation products, and decreased villus height and increased crypt depths [66]. Laudadio and collaborators [67] changed the crude protein (CP) content of the diet while maintaining amino acids requirement and observed that the reduction of CP decreased aerobic mesophilic bacteria and *E. coli* count in broiler excreta. Moreover, after digesta viscosity, a high crude protein diet is the secondary predisposing factor for necrotic enteritis, especially if animal ingredients are used [68,69]. Higher crude protein increases *Clostridium perfringens* in the ileum and ceca [69]. Moreover, feed formulated to have the same CP content (400 g/kg) but from different protein ingredients, showed different enumeration of Clostridium perfringens. Diet with meat/bone meal, fish meal, feather meal or potato protein produced higher *C. perfringens* colonization in broilers than corn gluten meal, soy or pea protein concentrates, or the control diet [70]. The increase in undigestible protein and unabsorbed amino acids arriving in the bottom of ileum and ceca may be the cause for the increase in these bacteria with the use of high crude protein levels or protein of difficult digestion. It has been observed that some amino acids in the intestine, such as methionine and glycine, stimulate *C. perfringens* growth [71,72]. Wilkie and collaborators [70] observed that the glycine content of the diets and ileal content were positively correlated with Clostridium perfringens count in ileum and ceca. As the glycine content of animal ingredients is 2 to 4 times higher relative to CP than vegetal protein source [73], it might be the supporter of the *Clostridium perfringens* growth and may be the reason for the higher content of the bacteria with animal diets. 

Therefore, the high-protein diet has been correlated with intestinal inflammation in humans, broilers eating diets with high CP can suffer similar effects on their GIT. Likewise, research in chickens has shown the undesirable effects of diets with high indigestible protein, high crude protein, and animal ingredients; these factors should be evaluated by nutritionists. When aiming for a healthy gut, diets should be formulated, while economically feasible, to have amino acids coming from supplementation, high digestible vegetal ingredients, and/or have proteases added to the feed.

### 4.4. Mycotoxins

Mycotoxins are secondary metabolites produced by fungi that contaminate grains mainly if the crops were poorly harvested or exposed to improper conditions during transportation up to marketing and use [74]. The ingestion of mycotoxins can induce systemic effects and generated what is called mycotoxicosis. Some of the toxic effects of mycotoxins are carcinogenic, mutagenic, estrogenic, hemorrhagic, immunotoxic, nephrotoxic, hepatotoxic, dermatoxic and neurotoxic [75]. However, a large number of papers focused on other systems effects; the GIT mucosa is the first animal tissue in contact with mycotoxins, it acts as a filter to these harmful toxins to the whole body but it suffers some of the mycotoxin’s toxic effects too [76].

Trichothecenes, as T-2 toxin (Type A) and deoxynivalenol (DON), are produced by *Fusarium graminearum* and their intoxication cause decrease absorption of glucose [76], and the reduction of villus height and ratio of villus height:crypt depth in broilers [77]. Also, a pro-inflammtory response in the small intestine was observed with the consumption of DON in swine, murine and human’s studies [78,79,80]. Fumonisin, produced by *Fusarium* sp., showed effects on intestinal cell lines reducing viability and proliferation [81] and suppressing tight junction protein expression [82].

The presence of ochratoxin A (OTA) in the feed increased the observation of macro-lesions in intestine and increased the animal susceptibility to *Eimeria* sp. [83] and *E. coli* O78 [84] infections. OTA also induces oxidative stress that might be the reason for negative findings such as the reduction of villus height: crypt depth ratio in broilers [85]. Solcan et al. [86] increased OTA concentrations in the broilers feed and observed modification of the architecture of intestinal epithelia, with a decrease of villus height:crypt depth ratio in the duodenum, necrosis areas, apoptosis, altered glands of lamina propria, and taller enterocytes with big and multiple nuclei and sometimes with no brush border. In the same study, when evaluating the lymphoid associated tissue, TCR1, TCR2, CD4+ and CD8+ intraepithelial lymphocytes in epithelia were reduced and showed death signs as pyknosis and cortical hyperchromatosis. However, in the lamina propria, the number of CD4+ and CD8+ was higher in animals exposed to OTA. 

Aflatoxin is the most common mycotoxin contamination in animal feed. Aflatoxin B1 (AFB1) is listed as the group I carcinogen by Agency for Research on Cancer (IARC) and one of the most potent hepatocarcinogens to mammals [87]. In vitro experiments with human colon cells (Caco-2) showed that AFB1 inhibits cell growth, increases lactate dehydrogenase activity and produces genetic damage [88]. Compared to other mycotoxins, such fumonisin and DON, aflatoxin B1 is highly absorbed by poultry intestine (>80%) mainly in the upper gut [89]. Even with the fast absorption, aflatoxin B1 showed detrimental effects on the gut epithelia in broilers, increasing gut permeability, reducing apparent ileal digestible energy, and reducing standardized nitrogen and amino acids digestibility [90]. When broilers were fed with AFB1 contamined diets, birds showed a reduction in the intestinal density [91]. Diet with 1 mg AFB1/kg for 4 weeks promoted necrosis in the crop, and catarrhal enteritis with lymphocytic or mononuclear cell infiltrations in the intestine of chickens [84].

Therefore, despite the systemic effects of mycotoxins, they also might affect the gastrointestinal tract, triggering inflammation, increasing gut permeability, apoptosis, and reducing digestibility, as well as, increasing the susceptibility of animals to intestinal pathologies. Thus, these factors prove the importance of crop management, storage, and overall grain quality for the maintenance of a healthy and functional GIT. In some cases of mycotoxins contaminations, the use of adsorbents can be helpful do diminish the harmful effects of mycotoxins. 

## 5. Conclusions and Perspectives

Some dietary components might be a challenge for the maintenance of gut homeostasis, but they can be easily manipulated. Thus, it is vital to know how dietary components interact with the intestinal immune system. For example, feed with high content of non-starch polysaccharides, crude protein, rancid ingredients, or contaminated with mycotoxins has been shown to trigger inflammation on the gut of poultry species. Even in small quantities, these dietary components can be detrimental to intestinal health. They can act as a chronic inflammatory trigger, as a result of the injuries they cause and a constant daily exposure to the gut epithelium. Therefore, understanding how dietary ingredients can affect the intestine immunity is important for poultry gut health and production in the future. Furthermore, exogenous enzymes, pro and pre-biotics, antioxidants, and adsorbents are some of the additives that can be wisely used to help maintain proper gut health of a flock and diminish any low-grade dietary chronic inflammation caused by the feed components.

## References

[1] Lee K.W., Lee S.H., Lillehoj H.S., Li G.X., Jang S.I., Babu U.S., Park M.S., Kim D.K., Lillehoj E.P., Neumann A.P. (2010). Effects of direct-fed microbials on growth performance, gut morphometry, and immune characteristics in broiler chickens. Poult. Sci..

[2] Kogut M.H., Genovese K.J., Swaggerty C.L., He H., Broom L. (2018). Inflammatory phenotypes in the intestine of poultry: not all inflammation is created equal. Poult. Sci..

[3] Teirlynck E., Bjerrum L., Eeckhaut V., Huygebaert G., Pasmans F., Haesebrouck F., Dewulf J., Ducatelle R., Van Immerseel F. (2009). The cereal type in feed influences gut wall morphology and intestinal immune cell infiltration in broiler chickens. Br. J. Nutr..

[4] Teirlynck E., Gussem M.D.E., Dewulf J., Haesebrouck F., Ducatelle R., Van Immerseel F. (2011). Morphometric evaluation of “dysbacteriosis” in broilers. Avian Pathol..

[5] Niewold T.A. (2007). The Nonantibiotic Anti-Inflammatory Effect of Antimicrobial Growth Promoters, the Real Mode of Action? A Hypothesis. Poult. Sci..

[6] Phillips I., Casewell M., Cox T., De Groot B., Frits C., Jones R., Nightingle C., Preston R., Waddell J. (2004). Does the use of antibiotics in food animals pose a risk to human health? A critical review of published data. J. Antimicrob. Chemother.

[7] Brewer M.S., Rojas M. (2008). Consumer attitudes towards issues in food safety. J. Food Saf..

[8] Phillips I. (2007). Withdrawal of growth-promoting antibiotics in Europe and its effects in relation to human health. Int. J. Antimicrob. Agents.

[9] Smith J.A. (2011). Experiences with drug-free broiler production. Poult. Sci..

[10] Morgan N.K. (2017). Managing gut health without reliance on antimicrobials in poultry. Anim. Prod. Sci..

[11] Oviedo-Rondón E.O. (2019). Holistic view of intestinal health in poultry. Anim. Feed Sci. Technol..

[12] Cummings J.H., Antoine J.M., Azpiroz F., Bourdet-Sicard R., Brandtzaeg P., Calder P.C., Shortt C. (2004). PASSCLAIM- Gut health and immunity. Eur. J. Nutr..

[13] Kogut M.H., Arsenault R.J. (2016). AMPK and mTOR: sensors and regulators of immunometabolic changes during Salmonella infection in the chicken. Poult. Sci..

[14] Broom L.J., Kogut M.H. (2017). Inflammation: friend or foe for animal production?. Poult. Sci..

[15] Jiminez J.A., Uwiera T.C., Douglas Inglis G., Uwiera R.R. (2015). Animal models to study acute and chronic intestinal inflammation in mammals. Gut Pathog..

[16] Kogut M.H. (2013). The gut microbiota and host innate immunity: Regulators of host metabolism and metabolic diseases in poultry?. J. Appl. Poult. Res..

[17] Nishio J., Honda K. (2012). Immunoregulation by the gut microbiota. Cell. Mol. Life Sci..

[18] Buffie C.G., Pamer E.G. (2013). Microbiota-mediated colonization resistance against intestinal pathogens. Nat. Rev. Immunol..

[19] Lawley T.D., Walker A.W. (2013). Intestinal colonization resistance. Immunology.

[20] Chovatiya R., Medzhitov R. (2014). Stress, inflammation, and defense of homeostasis. Mol. Cell.

[21] Kogut M.H. (2017). Issues and consequences of using nutrition to modulate the avian immune response. J. Appl. Poult. Res..

[22] Broom L.J. (2019). Host-microbe interactions and gut health in poultry—Focus on innate responses. Microorganisms.

[23] Keestra A.M., de Zoete M.R., Bowman L.T., Vaezirod M.M., van Putten J.P.M. (2013). Unique features of chicken tolllike receptors. Dev. Comp. Immunol..

[24] Smith A.L., Powers C., Beal R.K., Shat K.A., Kaspers B., Kaiser P. (2014). The avian enteric immune system in health and disease. Avian Immunology.

[25] Wu J., Chen Z.J. (2014). Innate immune sensing and signaling of cytosolic nucleic acids. Annu. Rev. Immunol..

[26] Fiocchi C. (2008). What is “physiological” intestinal inflammation and how does it differ from “pathological” inflammation?. Inflamm. Bowel Dis..

[27] Assmann N., Finlay D.K. (2016). Metabolic regulation of immune responses; therapeutic opportunities. J. Clin. Investig..

[28] Lackey D.E., Olefsky J. (2016). Regulation of metabolism by the innate immune system. Nat. Rev. Endocrinol..

[29] Gregor M.F., Hotamisligil G.S. (2011). Inflammatory mechanisms in obesity. Annu. Rev. Immunol..

[30] Rubartelli A., Lotze M.T., Latz E., Manfredi A. (2013). Mechanisms of sterile inflammation. Front. Immunol..

[31] Williams J.M., Duckworth C.A., Burkitt M.D., Watson A.J.M., Campbell B.J., Pritchard D.M. (2015). Epithelial cell shedding and barrier function. Vet. Pathol..

[32] Chen J., Tellez G., Richards J.D., Escobar J. (2015). Identification of potential biomarkers for gut barrier failure in broiler chickens. Front. Vet. Sci..

[33] Awad W.A., Hess C., Hess M. (2017). Enteric pathogens and their toxin induced disruption of the intestinal barrier through alteration of tight junctions in chickens. Toxins.

[34] Song J., Xiao K., Ke Y.L., Jiao L.F., Hu C.H., Diao Q.Y., Shi B., Zou X.T. (2014). Effect of a probiotic mixture on intestinal microflora, morphology, and barrier integrity of broilers subjected to heat stress. Poult. Sci..

[35] Jiang Z., Schatzmayr G., Mohnl M., Applegate T.J. (2010). Net effect of an acute phase response—Partial alleviation with probiotic supplementation. Poul. Sci..

[36] Arsenault R.J., Lee J.T., Latham R., Carter B., Kogut M.H. (2017). Changes in immune and metabolic gut response in broilers fed β-mannanase in β-mannan-containing diets. Poult. Sci..

[37] Bailey R.W., Butler G.W. (1973). Chemistry and Biochemistry of Herbage.

[38] Johansson L., Tuomainen P., Ylinen M., Ekholm P., Virkki L. (2004). Structural analysis of water-soluble and-insoluble β-glucans of whole-grain oats and barley. Carbohydr. Polym..

[39] Knudsen K.B. (2001). The nutritional significance of “dietary fibre” analysis. Anim. Feed Sci. Technol..

[40] Bederska-Łojewska D., Świątkiewicz S., Arczewska-Włosek A., Schwarz T. (2017). Rye non-starch polysaccharides: their impact on poultry intestinal physiology, nutrients digestibility and performance indices–a review. Ann. Anim. Sci..

[41] Boros D., Marquardt R.R., Guenter W. (1998). Site of exoenzyme action in gastrointestinal tract of broiler chicks. Can. J. Anim. Sci..

[42] Fuller R. (2001). The chicken gut microflora and probiotic supplements. J. Poult. Sci..

[43] Józefiak D., Rutkowski A., Martin S.A. (2004). Carbohydrate fermentation in the avian ceca: a review. Anim. Feed Sci. Technol..

[44] Cardoso V., Ferreira A.P., Costa M., Ponte P.I.P., Falcão L., Freire J.P., Ribeiro T. (2014). Temporal restriction of enzyme supplementation in barley-based diets has no effect in broiler performance. Anim. Feed Sci. Technol..

[45] Slominski B.A. (2011). Recent advances in research on enzymes for poultry diets. Poult. Sci..

[46] Józefiak D., Rutkowski A., Jensen B.B., Engberg R.M. (2006). The effect of β-glucanase supplementation of barley-and oat-based diets on growth performance and fermentation in broiler chicken gastrointestinal tract. Br. Poult. Sci..

[47] Hashemipour H., Khaksar V., Rubio L.A., Veldkamp T., Van Krimpen M.M. (2016). Effect of feed supplementation with a thymol plus carvacrol mixture, in combination or not with an NSP-degrading enzyme, on productive and physiological parameters of broilers fed on wheat-based diets. Anim. Feed Sci. Technol..

[48] Latorre J.D., Hernandez-Velasco X., Bielke L.R., Vicente J.L., Wolfenden R., Menconi A., Tellez G. (2015). Evaluation of a Bacillus direct-fed microbial candidate on digesta viscosity, bacterial translocation, microbiota composition and bone mineralisation in broiler chickens fed on a rye-based diet. Br. Poult. Sci..

[49] Tellez G., Latorre J.D., Kuttappan V.A., Hargis B.M., Hernandez-Velasco X. (2015). Rye affects bacterial translocation, intestinal viscosity, microbiota composition and bone mineralization in turkey poults. PLoS ONE.

[50] Choct M. (2015). Feed non-starch polysaccharides for monogastric animals: classification and function. Anim. Prod. Sci..

[51] Latorre J.D., Hernandez-Velasco X., Kuttappan V.A., Wolfenden R.E., Vicente J.L., Wolfenden A.D., Tellez G. (2015). Selection of Bacillus spp. for cellulase and xylanase production as direct-fed microbials to reduce digesta viscosity and Clostridium perfringens proliferation using an in vitro digestive model in different poultry diets. Front. Vet. Sci..

[52] Chen J.R., Chen Y.L., Peng H.C., Lu Y.A., Chuang H.L., Chang H.Y. (2016). Fish oil reduces hepatic injury by maintaining normal intestinal permeability andmicrobiota in chronic ethanol-fed rats. Gastroent. Res. Pract..

[53] Yangilar F. (2016). Effect of the fish oil fortified chitosan edible film on microbiological, chemical composition and sensory properties of Göbek Kashar Cheese during ripening time. Korean. J. Food. Sci. Anim. Resour..

[54] Hui Y.H. (2001). Bailey’s industrial oil and fat products. J. Nutr..

[55] Shibamoto T. (2006). Analytical methods for trace levels of reactive carbonyl compounds formed in lipid peroxidation systems. J. Pharm. Biomed. Anal..

[56] Dibner J.J., Atwell C.A., Kitchell M.L., Shermer W.D., Ivey F.J. (1996). Feeding of oxidized fats to broilers and swine: effects on enterocyte turnover, hepatocyte proliferation and the gut associated lymphoid tissue. Anim. Feed Sci. Technol..

[57] Kubow S. (1990). Toxicity of dietary lipid peroxidation products. Trends Food Sci. Technol..

[58] Liang F., Jiang S., Mo Y., Zhou G., Yang L. (2015). Consumption of Oxidized Soybean Oil Increased Intestinal Oxidative Stress and Affected Intestinal Immune Variables in Yellow-feathered Broilers. Asian Australas. J. Anim. Sci..

[59] Tan L., Rong D., Yang Y., Zhang B. (2018). The Effect of Oxidized Fish Oils on Growth Performance, Oxidative Status, and Intestinal Barrier Function in Broiler Chickens. J. Appl. Poult. Res..

[60] Açıkgöz Z., Bayraktar H., Altan Ö., Akhisaroglu S.T., Kırkpınar F., Altun Z. (2011). The effects of moderately oxidised dietary oil with or without vitamin E supplementation on performance, nutrient digestibility, some blood traits, lipid peroxidation and antioxidant defence of male broilers. J. Sci. Food Agric..

[61] Neuman M.G., Nanau R.M. (2012). Inflammatory bowel disease: role of diet, microbiota, life style. Transl. Res..

[62] Conlon M.A., Bird A.R. (2015). The Impact of Diet and Lifestyle on Gut Microbiota and Human Health. Nutrients.

[63] Silvester K.R., Cummings J.H. (1995). Does digestibility of meat protein help explain large-bowel cancer risk. Nutr. Cancer.

[64] Hughes R., Magee E.A., Bingham S. (2000). Protein degradation in the large intestine: Relevance to colorectal cancer. Curr. Issues Intest. Microbiol..

[65] Toden S., Bird A.R., Topping D.L., Conlon M.A. (2005). Resistant starch attenuates colonic DNA damage induced by higher dietary protein in rats. Nutr. Cancer.

[66] Qaisrani S.N., Moquet P.C.A., van Krimpen M.M., Kwakkel R.P., Verstegen M.W.A., Hendriks W.H. (2014). Protein source and dietary structure influence growth performance, gut morphology, and hindgut fermentation characteristics in broilers. Poult. Sci..

[67] Laudadio V., Dambrosio A., Normanno G., Khan R.U., Naz S., Rowghani E., Tufarelli V. (2012). Effect of Reducing Dietary Protein Level on Performance Responses and some Microbiological Aspects of Broiler Chickens under Summer Environmental Conditions. Avian Biol. Res..

[68] Dahiya J.P., Wilkie D.C., Van Kessel A.G., Drew M.D. (2006). Potential strategies for controlling necrotic enteritis in broiler chickens in post-antibiotic era. Anim. Feed Sci. Technol..

[69] Drew M.D., Syed N.A., Goldade B.G., Laarveld B., Van Kessel A.G. (2004). Effects of Dietary Protein Source and Level on Intestinal Populations of Clostridium perfringens in Broiler Chickens. Poult. Sci..

[70] Wilkie D.C., Van Kessel A.G., White L.J., Laarveld B., Drew M.D. (2005). Dietary amino acids affect intestinal Clostridium perfringens populations in broiler chickens. Can. J. Anim. Sci..

[71] Muhammed S.I., Morrison S.M., Boyd W.L. (1975). Nutritional requirements for growth and sporulation of Clostridium perfringens. J. Appl. Bacteriol..

[72] Ispolatovskaya M.V., Kadis A.S., Montie T.C., Ajl S.J. (1971). Type a Clostridium perfringens toxin. Microbial Toxins.

[73] AminoDat^TM^ (2001). Degussa Feed Additives.

[74] Khazaeli P., Najafi M.L., Bahaabadi G.A., Shakeri F., Naghibzadeh tahami A. (2014). Evaluation of aflatoxin contamination in raw and roasted nuts in consumed Kerman and effect of roasting, packaging and storage conditions. Life Sci. J..

[75] Milićević D.R., Škrinjar M., Baltić T. (2010). Real and perceived risks for mycotoxin contamination in foods and feeds: challenges for food safety control. Toxin.

[76] Liew W.P.P., Mohd-Redzwan S. (2018). Mycotoxin: Its Impact on Gut Health and Microbiota. Front. Cell. Infect. Microbiol..

[77] Yu Y.H., Hsiao F.S.H., Proskura W.S., Dybus A., Siao Y.H., Cheng Y.H. (2018). An impact of Deoxynivalenol produced by Fusarium graminearum on broiler chickens. J. Anim. Physiol. Anim. Nutr..

[78] Bracarense A.P.F., Lucioli J., Grenier B., Drociunas Pacheco G., Moll W.-D., Schatzmayr G., Oswald I.P. (2012). Chronic ingestion of deoxynivalenol and fumonisin, alone or in interaction, induces morphological and immunological changes in the intestine of piglets. Br. J. Nutr..

[79] Li M., Cuff C.F., Pestka J. (2005). Modulation of murine host response to enteric reovirus infection by the trichothecene deoxynivalenol. Toxicol. Sci..

[80] Maresca M., Yahi N., Younès-Sakr L., Boyron M., Caporiccio B., Fantini J. (2008). Both direct and indirect effects account for the pro-inflammatory activity of enteropathogenic mycotoxins on the human intestinal epithelium: Stimulation of interleukin-8 secretion, potentiation of interleukin-1β effect and increase in the transepithelial passage of commensal bacteria. Toxicol. Appl. Pharmacol..

[81] Minervini F., Garbetta A., D’Antuono I., Cardinali A., Martino N.A., Debellis L., Visconti A. (2014). Toxic mechanisms induced by fumonisin B1 mycotoxin on human intestinal cell line. Arch. Environ. Contam. Toxicol..

[82] Romero A., Ares I., Ramos E., Castellano V., Martínez M., Martínez-Larrañaga M.R., Martínez M.A. (2016). Mycotoxins modify the barrier function of Caco-2 cells through differential gene expression of specific claudin isoforms: protective effect of illite mineral clay. Toxicology.

[83] Manafi M.K.M., Noor Ali M. (2011). Effect of ochratoxin A on coccidiosis-challenged broiler chicks. Effect of ochratoxin A on coccidiosis-challenged broiler chicks. World Mycotoxin J..

[84] Kumar A., Jindal N., Shukla C.L., Pal Y., Ledoux D.R., Rottinghaus G.E. (2003). Effect of Ochratoxin A on Escherichia coli–Challenged Broiler Chicks. Avian Dis..

[85] Qu D., Huang X., Han J., Man N. (2017). Efficacy of mixed adsorbent in ameliorating ochratoxicosis in broilers fed ochratoxin A contaminated diets. Ital. J. Anim. Sci..

[86] Solcan C., Pavel G., Floristean V., Chiriac I., Şlencu B., Solcan G. (2015). Effect of ochratoxin A on the intestinal mucosa and mucosa-associated lymphoid tissues in broiler chickens. Acta Vet. Hung..

[87] Muhammad I., Sun X., Wang H., Li W., Wang X., Cheng P., Hamid S. (2017). Curcumin successfully inhibited the computationally identified CYP2A6 enzyme-mediated bioactivation of aflatoxin B1 in arbor acres broiler. Front. Pharmacol..

[88] Zhang J., Zheng N., Liu J., Li F., Li S., Wang J. (2015). Aflatoxin B1 and aflatoxin M1 induced cytotoxicity and DNA damage in differentiated and undifferentiated Caco-2 cells. Food Chem. Toxicol..

[89] Grenier B., Applegate T.J. (2013). Modulation of intestinal functions following mycotoxin ingestion: meta-analysis of published experiments in animals. Toxins.

[90] Chen X., Naehrer K., Applegate T.J. (2016). Interactive effects of dietary protein concentration and aflatoxin B1 on performance, nutrient digestibility, and gut health in broiler chicks. Poult. Sci..

[91] Hossein A., Gürbüz Y. (2016). Aflatoxins in Poultry Nutrition. J. Nat. Sci..

